# 

**Published:** 1898-02-19

**Authors:** 


**Notices of Births, Deaths, and Marriages** are inserted in this column at a charge of 2s. 6d. for an announcement not exceeding four lines.

**Marriage.**

Hargreaves—Jessop.—On December 18th, at St. Michael's and All Angels, Christchurch, New Zealand, by the Rev. A. W. Averill, M.A., Walter Herbert Hargreaves, M.R.C.S., L.R.C.P.London, of Akaroa, N.Z., to Alice, third daughter of Z. Jessop, Esq., The Park, Nottingham, England. (1,404)

### NOTICE TO CORRESPONDENTS.

**All MS., Letters, Books for Review, and other matters intended for the Editor should be addressed** The Editor, "The Hospital," The Hospital Building, 28 & 29, Southampton Street, Strand, W.C.

**The Editor cannot undertake to return rejected MS., even when accompanied by stamped directed envelope.**

**Notice to Advertisers and Subscribers.**

**All Advertisements, Orders for Copies of** The Hospital, **and Business Communications should be addressed to THE MANAGER, (Not to The Editor),** The Hospital, The Hospital Building, 28 & 29, Southampton Street, Strand, London, W.C.

**The Cost of Subscription, post free, is as follows:—Per week, 2½d. per quarter, 2s. 8d.; per half-year, 5s. 3d.; per annum, 10s. 6d. Foreign:—Per annum, 15s. 2d.: payable, in all cases, in advance.**

**NOTICE.—Vol. XXII. of "The Hospital" (April, 1897, to September, 1897), in handsome cloth binding, is now on sale at the Office of this Journal, price 7s. 6d. Cases for binding the Numbers contained in this Volume. 1s. 6d. Index to Vol. XXII., 2½d., post free. The Monthly Part for March will be ready on March 1st. Price 6d., by post 8d.**

### The Student of Medicine.

### APPOINTMENTS.

W. J. Beatty, L.R.C.P.Edin., L.M., L.F.P.S.Glasg., J.P., appointed Certifying Factory Surgeon for the Stockton District. J. Carroll, M.B., C.M.Glasg., D.P.H.Camb., appointed Lecturer on Hygiene and Public Health to the Anderson's College School of Medicine, Glasgow. J. Fitzgerald, B.A., M.B., C.M., appointed House Surgeon to the Edinburgh Royal Maternity and Simpson Memorial Hospital. G. R. Harland, L.R.C.P., L.R.C.S.Edin., appointed Assistant Medical Officer of the Workhouse of the Gateshead Union. H. Horrocks, appointed Assistant Pathologist and Bacteriologist at the Public Hospital, Perth, West Australia. Dr. Mackintosh, reappointed Medical Officer of Health to the Clay Cross Urban District Council. C. P. Mathew, L.R.C.P.Lond., M.R.C.S.Eng., appointed Medical Officer for the Hatherleigh District of the Okehampton Union. R. C. B. Maunsell, M.B., B.Ch.Dub., appointed Surgeon to the Mercers' Hospital, Dublin. G. V. Miller, M.B., C.M.Edin., appointed Ophthalmic Surgeon to the Northallerton Hospital. A. D. Owen, B.Sc., M.R.C.S., L.R.C.P.Lond., appointed Government Surgeon at Enkeldoorn, Rhodesia, South Africa. E. E. Porritt, M.B., C.M., appointed House Surgeon to the Edinburgh Royal Maternity and Simpson Memorial Hospital. J. P. Race, L.S.A.Lond., appointed Junior House Surgeon to the Ancoats Hospital, Manchester.

### ARMY MEDICAL STAFF CORPS.

The following gentlemen successfully passed the examinations recently held for commissions in the Army Medical Staff. The names are placed in order of merit: W. H. S. Nickerson, A. E. Walter, G. S. Nickerson, A. E. Weld, J. S. Gallie, G. R. Crisp, H. B. G. Walton, W. Jagger, A. B. MacCarthy, R. Selby, A. E. Thorp, E. J. Dobbin, A. R. O'Flahertie, H. Herrick, C. W. Mainprise, G. J. S. Archer, R. S. H. Fuhr, H. O. Hall, F. J. C. Heffernan, J. Cowan, and E. P. Hewitt.

